# Supplementary material for: Population characteristics, PrEP eligibility, and trust in family planning providers among women accessing public family planning clinics in Kenya
Source: PLOS Glob Public Health. 2026 Feb 26;6(2):e0005480. doi: 10.1371/journal.pgph.0005480 (PMC12944804; doi:10.1371/journal.pgph.0005480)
Supplement: S1 Text — (DOCX) [file pgph.0005480.s001.docx]

**Table A: Demographic Characteristics of Women Accessing Family Planning Services, stratified by Clinic Location**

| **Covariates** | **🞰Urban clinic (N=608)** | **Semi-Urban**  **(N=887)** | **Rural clinics**  **(N=306)** |
| --- | --- | --- | --- |
|  | **n/N (%) or Median (IQR)** | **n/N (%) or Median (IQR)** | **n/N (%) or Median (IQR)** |
| **Demographics** |  |  |  |
| **Age in years, Median (Q1-Q3)** | 27.2 (23.6-32.0) | 26.3 (23.0-31.0) | 28.0 (23.4-33.0) |
| **Age Category** |  |  |  |
| 15-19 | 22/608 (3.62) | 71/887(8.00) | 24/306 (7.84) |
| 20-24 | 19/608 1(31.41) | 275/887 (31.00) | 82/306 (26.80) |
| 25-34 | 297/608 (48.85) | 423/887 (47.69) | 145/306 (47.39) |
| ≥35 | 95/608 (15.63) | 118/887 (13.30) | 54/306 (17.65) |
| Missing | 3/608 (0.49) | 0/887 (0.00) | 0/306 (0.00) |
| **Marital status** |  |  |  |
| Married | 485/608 (79.77) | 679/887 (76.55) | 237/306 (77.45) |
| Not Married | 105/608 (17.27) | 199/887 (22.44) | 69/306 (22.55) |
| Not Applicable/Unknown | 18/608 (2.96) | 9/887 (1.01) | 0/306 (0.00) |
| **Education** |  |  |  |
| Primary and below | 73/608 (12.01) | 148/887 (16.69) | 76/306 (24.84) |
| Secondary Education | 320/608 (52.63) | 566/887 (63.81) | 193/306 (63.07) |
| Attended post-secondary school | 197/608 (32.40) | 164/887 (18.49) | 37/306 (12.09) |
| Unknown | 18/608 (3.0) | 9/887 (1.01) | 0/306 (0.00) |
| **Occupation** |  |  |  |
| Employed (Formal/Informal) | 364/608(59.9) | 439/887 (49.5) | 134/306 (43.8) |
| Unemployed | 115/608 (19.01) | 198/887 (22.3) | 82/306 (26.8) |
| Student Status | ^71/608 (11.74)^ | 127/887 (14.3) | 30/306 (9.8) |
| Household Wife/Child Care | ^39/608 (6.4)^ | 114(/88712.9) | 60/306 (19.6) |
| Missing | ^19/608 (3.1)^ | 9/887 (1.0) |  |
| **Personal Income** |  |  |  |
| No Income | 219/608 (36.0) | 397/887 (44.8) | 161/306 (52.6) |
| 1 - 5,000 Ksh | 89/608 (14.6) | 83/887 (9.4) | 33/306 (10.8) |
| 5,001 - 10,000 Ksh | 131/608 (21.5) | 134/887 (15.1) | 55/306 (18.0) |
| >10,000 ksh | 108(/608 17.8) | 131/887 (14.8) | 38/306 (12.4) |
| Declined Response/Unknown | 61/608 (10.0) | 142/887 (16.0) | 19/306 (6.2) |
| **Reason for visiting FP clinic*** |  |  |  |
| Family planning | 588/605 (97.2) | 867/887 (97.7) | \| 300/306 (98.0) \| \| --- \| |
| HIV testing or Prevention/ STI testing or Treatment | 71/605 (11.7) | 158/887 (17.8) | \| 35/306 (11.4) \| \| --- \| |
| Cervical cancer screening | 32/605 (5.3) | 70 /887 (7.9) | \| 24/306 (7.8) \| \| --- \| |
| Others | 97/605 (16.0) | 141/887 (15.9) | \| 38/306 (12.4) \| \| --- \| |
| **Number of times visited FP clinic in last 12 months** |  |  |  |
| First visit today | 254/608 (41.8) | 302/887 (34.0) | 90/306 (29.4) |
| 2-3 visits | 282/608 (46.4) | 479/887 (54.0) | 191/306 (62.4) |
| 4 or more visits | 69/608 (11.3) | 106/887 (12.0) | 25/306 (8.2) |
| Unknown | 3/306(0.5) | 0/887 (0.0) | 0/306 (0.00) |
| **Access to Contraception** |  |  |  |
| **Any Modern Contraception** |  |  |  |
| **Yes** | 445/608 (73.2) | 609/887 (68.7) | 243/306 (79.4) |
| **None** | 36(/608 5.9) | 53/887 (6.0) | 5/306 (1.6) |
| **Missing** | 127/608 (20.9) | 225/887 (25.4) | 127/306 (41.5) |
| **Current FP Method*** |  |  |  |
| Implant | 9/608 (1.5) | 165/887 (18.6) | 48/306 (15.7) |
| Injectable | 10/608 (1.6) | 202/887 (22.8) | 83/306 (27.1) |
| OCP | 10/608 (1.6) | 3/887 (0.34) | 3/306 (0.98) |
| IUCD | 4/608 (0.7) | 25/887 (2.8) | 7/306 (1.6) |
| Condoms and Sterilization | 8/608 (1.3) | 5/887 (0.6) | 0/306 (0.00) |
| None | 38/608 (6.3) | 53/887 (6.0) | 5/306 (2.3) |
| Missing | 556/608 (91.45) | 744/887 (83.9) | 290/306 (94.8) |
| **Received Preferred FP method** |  |  |  |
| Yes | 334/306 (54.9) | 484/887 (54.6) | 49/306 (16.0) |
| No/ Not sure | 31/608 (5.1) | 110/887 (12.4) | 14/306 (4.6) |
| Not Applicable/ didn't receive a method | 334/608 (54.9) | 293/887 (33.0) | 49/306 (16.0) |
| **What they would have wanted to receive*** |  |  |  |
| Implant | 9/608 (1.48) | 51/887 (5.7) | \| 6/306 (1.96) \| \| --- \| |
| Injectable | 10/608 (1.64) | \| 59/887 (6.7) \| \| --- \| \|  \| | \| 5/306 (1.6) \| \| --- \| |
| OCP | 10/608 (1.64) | 3/887 (0.3) | 3/306(0.98) |
| IUCD | 4/608 (0.65) | 1/887 (0.6) | \| 1/306 (0.33) \| \| --- \| |
| Others | 8/608 (1.32) | 5/887 (0.6) | 0/306 (0.00) |
| None | 11/608 (1.80) | \| 24/887 (2.7) \| \| --- \| \|  \| | \| 1/306 (0.33) \| \| --- \| |
| Missing | 553/608 (90.0) | 744/887 (83.4) | \| 289/106 (94.4) \| \| --- \| |
| **Overall Clinical Experience/Satisfaction** |  |  |  |
| Very Satisfied/Somewhat satisfied | 595/608 (97.9) | 803/887 (90.5) | 288/306 (94.1) |
| Totally Dissatisfied/Somewhat dissatisfied | 10/608 (1.6) | 84/887 (9.5) | 18/306 (5.9) |
| Unknown | 3/608 (0.5) | 0/887 (0.0) | 0/306 (0.00) |

**🞰** **We included 4 participants with missing age data. The age stratified analysis comprises 1,797 participants instead of 1801 participants:** $\boldsymbol{*}$**Multiple choice selection; OCP: Oral Contraceptive Pills; IUCD: Intrauterine Contraceptive Device.**

**Table B: Distribution of participants stratified by Clinic Sites**

| **Clinic Site Name** | **N=1801** |
| --- | --- |
|  | **n/N (%)** |
| A | 373/1801 (20.71) |
| B | 88/1801 (4.89) |
| C | 95/1801 (5.27) |
| D | 164/1801 (9.11) |
| E | 193/1801 (10.72) |
| F | 64/1801 (3.55) |
| G | 251/1801 (13.94) |
| H | 68/1801 (3.78) |
| I | 207/1801 (11.49) |
| J | 76/1801 (4.22) |
| K | 98/1801 (5.44) |
| L | 124/1801 (6.89) |
|  |  |
