# Supplementary material for: Population characteristics, PrEP eligibility, and trust in family planning providers among women accessing public family planning clinics in Kenya
Source: PLOS Glob Public Health. 2026 Feb 26;6(2):e0005480. doi: 10.1371/journal.pgph.0005480 (PMC12944804; doi:10.1371/journal.pgph.0005480)
Supplement: Attachment — Submitted filename: Response to Editorial Team and Reviewers.docx [file pgph.0005480.s003.docx]

Dr. Andrew Kazibwe

Academic Editor

PLOS Global Public Health

RE: Submission ID: [PGPH-D-25-02465] - [EMID:ea28ca1fa7b6ae4b]

Dear Dr Andrew, Kazibwe,

On behalf of my co-authors, we are grateful for your valuable suggestions and recommendations and for the opportunity to revise and improve our study. Our manuscript has benefitted tremendously from these comments.

Below, we have attached a point-by-point response to each review suggestion, comment, and recommendation in *Italics*.

Thank you for allowing us to resubmit our manuscript for your consideration.

We look forward to hearing from you.

Sincerely,

David Mukasa, BVM, MVPM, PhD, MS, FRSPH

Assistant Teaching Professor, Department of Global Health

School of Public Health and School of Medicine

University of Washington

3980 15th Ave NE, Seattle, WA 98105

Phone: +1 (206) 685-2329

Email: [mukasa@uw.edu](mailto:mukasa@uw.edu)

**Response to Editorial Team and Reviewers

Journal Requirements:**

1. In the ethics statement in the Methods, you have specified that verbal consent was obtained. Please provide additional details regarding how this consent was documented and witnessed, and state whether this was approved by the IRB.

*Response: This exit survey was nested within a routine nation PrEP program as part of quality improvement. Consistent with routine care in these clinics only verbal consenting is given and document. This verbal consent was approved by the University of Washington Institution Review Board and a copy of the approval file titled “FP Plus Oral Consent Guide for CS Survey” was submitted together with the manuscript in the initial submission for review. We have resubmitted the file together with the revision and added the following sentence in the methods section in lines 191-193.*

*“Consistent with routine services and quality improvement, documentation of oral consenting for exit survey was waived by the IRB but the oral consent processing was based on an IRB approved oral consenting guide.”*

2. Please include a complete copy of PLOS’ questionnaire on inclusivity in global research in your revised manuscript. Our policy for research in this area aims to improve transparency in the reporting of research performed outside of researchers’ own country or community. The policy applies to researchers who have travelled to a different country to conduct research, research with Indigenous populations or their lands, and research on cultural artefacts. The questionnaire can also be requested at the journal’s discretion for any other submissions, even if these conditions are not met. Please find more information on the policy and a link to download a blank copy of the questionnaire here: [https://journals.plos.org/globalpublichealth/s/best-practices-in-research-reporting](https://urldefense.com/v3/__https:/journals.plos.org/globalpublichealth/s/best-practices-in-research-reporting__;!!K-Hz7m0Vt54!kg9eLj4ERkwo70D_sUUF_y62A0bnG97d5DJnx3K71MELX_CCV2UygCgdhMdHYhArX-MQqOCOJ2Urxg$). Please upload a completed version of your questionnaire as Supporting Information when you resubmit your manuscript.

*Response: A completed copy of the PLOS’ questionnaire on inclusivity in global research has now been submitted together with the revision.*

If the reviewer comments include a recommendation to cite specific previously published works, please review and evaluate these publications to determine whether they are relevant and should be cited. There is no requirement to cite these works unless the editor has indicated otherwise.

*Response: We thank the editorial team for this suggestion. There was no specific published work recommended by reviewers for citation*.

Additional Editor Comments (if provided):

Editor Query:
The corresponding author should submit a signed cover letter.
I request the authors to provide a table summarising number of participants by study site

*Response: Thank you. We provided a cover letter in the initial submission. We resubmitted the letter with the corresponding author’s signature as seen in the attached files. Related to the summary of* number of participants by clinics*, we have now provided these as part of supplementary information. However, we also want to clarify these were not clinical study research sites in the true sense but real-world public health clinics which women access for routine care. So, whereas we understand that knowledge of the number interviewed per clinic adds important context, we want to point out and ensure no untestable conclusions are made solely based on any potential clinic variation. This is because, for this exit survey, there was no apriori hypothesis/intervention to test. The scientific question for the exit survey was to descriptively characterize the “WHO” accesses FP clinics and their general experiences, on average.*

Check grammar in lines 43, 98, 128, 145, and 280

*Response:* *We thank the editorial team for this valuable review. We have revised the sentence that was previously containing line 43 and currently lines 37-39 to add “in the” as follows: “Overall, 1801 participants were interviewed, representing 9.9% of all women without HIV who accessed the twelve clinics participating in the 24-month study period”*

*We have also revised the sentence that was previously containing line 98 and currently line 93-95 to add “a” as follows: “Briefly, the FP Plus Project was a pragmatic stepped wedge cluster-randomized project to integrate systematic screening and offer of oral PrEP to women accessing twelve primary care FP clinics in Kenya.”*

*We have also revised the sentence that was previously containing line 128 and currently lines 133-134 to update the tense and plurality as follows: “We used quantitative exit surveys administered to consecutive women exiting FP clinics to obtain detailed information on characteristics of women and their experiences accessing FP clinics.”*

*We have also revised the sentence that was previously containing line 145 and currently line 150-151 as follows: “inconsistent or no condom use, having a sex partner(s) at high risk and whose HIV status is unknown”*

*We have also revised the sentence that was previously containing line 280 and now currently on lines 292-294 as follows: “Despite high awareness of oral PrEP (68.9%), there was limited uptake of daily oral PrEP at the clinic when first offered—mainly due to low perceived risk, the need for more time to consult partner/family, potential stigma, and concern about pill burden.”*

Define technical support as used in line 101 - how was this different from training? What nature of training was given to the providers - classroom/online/on-site, skills or knowledge-based? How long was the training?

*Response:* *We have added the following language to the text to clarify and define the details of the implementation strategies applied in the parent project—provider training and technical support. It now on lines 96-104 and reads “The training was facility-based, used the Kenyan Ministry of Health case-based interactive training curriculum, and delivered over a 2-weeks before each clinic transitioned to the intervention period. Refresher sessions were conducted every three to six months to account for staff turnover. Technical assistance consisted of ongoing coaching and mentoring of healthcare workers in family planning clinics on PrEP delivery. Technical advisors were study-dedicated nurses with training and experience in oral PrEP delivery. They visited each clinic once a week for the first 3 months and then once a month for 9 months until each clinic had received the intervention package for 12 months.”*

Who administered the exit interviews? Were the interviewers part of the service providers?

*Response: We thank the reviewer for this valuable review. The exit interviews were administered* *by trained Kenyan study staff and were approved by IRB as part of inform quality improvement activities.*

*We have updated the methods study design section in lines 107-14 as follows: “Within the program, we used cross-sectional quantitative exit surveys administered on random days and clinics by trained study staff to consecutive women as they exited care across 12 clinics.* *The overall goal of the exit survey was to characterize the profile of women accessing family planning clinics* *and their experiences, including their HIV risk behaviors, type and quality of services received, and satisfaction with services.* *The sequence for the specific random days and clinic where the exit surveys would be conducted was generated by Kenya-based a study data manager every 3 months without any restriction/stratification.”*

Provide tool for HIV risk assessment

*Response: We thank the reviewer for this valuable suggestion. The risk assessment tool is used a guide to initiate conversation and counseling about HIV risk and PrEP eligibility per the Kenya guidelines. It’s not intended to serve as a risk-scoring tool. We have attached it as a supplementary material.*

How were days for the interviews selected? How was randomness ensured in selection of days for data collection? How was this expected to influence study results? Would the results have been different if the data collection was done on purposively selected days such as FP clinic days?

*Response: We apologize for the lack of clarification on the how days and clinics for exit survey was conducted. It is hard to speculate what the findings would have been with purposively selected days. However, we do know that any form of random selection would be expected, on average, to minimize selection bias and provide rigor to the evaluation. To improve clarity, we have revised the manuscript in lines 109-114 as follows: We have now added the following clarifying language “Within the program, we used cross-sectional quantitative exit surveys administered on random days and clinics by trained study staff to consecutive women as they exited care across 12 clinics. The sequence for the specific random days and clinic where the exit surveys would be conducted was generated by Kenya-based a study data manager every 3 months without any restriction/stratification.”*

In table 1 age categorisations are redundant as row headers

*Response: We thank the reviewer for this review. We added these categories to provide more details regarding the age distribution of study participants. The two age rows present different descriptive statistics. The first row presents age as a continuous variable, and the second-row header is for the categorized age variable. We believe that presenting both under one row headers may confuse readers more than currently presented.*

Similarly, in tables 2 and 3, the age and PrEP eligibility disaggregation as column headers make interpretation of p-values cumbersome.

*Response: We thank you so much for this excellent comment. We stratified the presentation by age and PrEP eligibility as contextual and scientifically key variables for this evaluation, based on and informed by our experience in HIV prevention for this population and settings. For women in Africa, women below <25 years disproportionately shoulder the burden of new HIV infections, and it is very important and critical to understand profile and unique experiences in accessing FP clinics. Similarly, not all women accessing FP clinics may need PrEP (at least not all the time). Thus, stratification on PrEP eligibility and defining their unique profile and experience is only imperative to understand potential coverage when you plan to introduce any HIV prevention intervention in that platform. Specifically, it helps to define whether FP clinics are an appropriate platform to reach the right women with HIV prevention needs. Thus, respectfully, we ask to present data as intended. Importantly, we agree that the p-values are confusing and less meaningful with apriori hypothesis/intervention to test. We have thus removed the P-values from the tables since this study is majorly descriptive.*

**Reviewer #1:**
Well written manuscript overall. Large sample size enabling to draw conclusions. However, there are a few clarifications needed.
Lines 71-72 It will be helpful to give the statistics in the text, not only the citation, for ease of reading.

*Response:*

*We have revised the sentence and included the statistics in lines 66-67 as follows:*

*“Higher rates of HIV infection were reported for women under 25 years irrespective of the contraceptive method (3.52 to 4.66 per 100-woman years) (2).”*

Line 145 " inconsistent OR condom use. Probably this is a typo for OF

*Response: We thank the reviewer for catching this typo. We have made the correct to “inconsistent or no condom use” as see in line 150.*

Line 150 Was the questionnaire to assess the trust in staff a standard one from literature or otherwise? Was it piloted? Was this based on a documented theoretical framework?

We thank the reviewer for this excellent comment. Although the survey documented itself and we have *added it as a key limitation, all the items/questions on the survey were collated from psychometrically developed tools and validated for this setting. Importantly, we have used this tool and items in this setting and population in several of our multiple clinical and implementation studies (including the Partners Scale up in HIV care clinics, PrIYA program in MCH/FP) and are confident in its content validity. We have added the following sentences in lines 318-323.*

*“Secondly, although the survey documented itself, all its items were derived from psychometrically developed instruments that have been validated for use in this setting. Importantly, we have previously employed this tool and its components in several clinical and implementation studies within the same population, such as the Partners Scale-Up in HIV care clinics and the PrIYA program in MCH/FP, which strengthens our confidence in its content validity”*

Line 155 ...whether they on or offered PrEP...Is the verb "were" missing here?

*We thank the reviewer for this valuable review. We have revised the sentence in lines previously 154-156 and currently 160-62 as follows “Additional themes assessed were whether they were on or offered PrEP, and if eligible but declined PrEP, reasons for declining /discontinuing PrEP, awareness and preference of different PrEP options.”*

Line 267 "unwanted pregnancy". Unplanned pregnancy is the preferred term, as a pregnancy occurring without adequate protection is expected. Moreover, women living with HIV have healthy babies with adequate prophylaxis. The sentence in the manuscript implies otherwise.

*Response: We thank the reviewer for this valuable suggestion. However, after reframing the paragraph, we deleted the sentence.*

Line 289 It is of interest to know if long-lasting PrEP is available and subsidised in the country

*Response: We thank the reviewer for this valuable suggestion. Currently, although bi-monthly injectable cabotegravir and monthly dapivirine vagina rings are approved by the Kenya Ministry of health, they are not yet available in Kenya as part of standard of care. Assessment of PrEP options preferences was based on a hypothetical scenario of future emerging options.*

Lines 297, 300 Could the authors clarify the terms used: randomized trial (implying comparison group) or cross-sectional study with random sampling methods. These are to distinctive study designs.
In-text: It would be interesting to know the reasons clients were dissatisfied with their clinic experience and had a lack of confidence in FP clinic staff. What theoretical framework informed measurement of patient satisfaction?

*Response: We thank the reviewer for this valuable suggestion. The cross-sectional study design is the study design for the current study, but this study was nested within a pragmatic stepped wedge cluster randomized trial as described in lines 90-95 as follows:*

*“Between July 26, 2021, and July 07, 2024, we conducted a cross-sectional study nested within a large pragmatic trial to integrate PrEP delivery in public sector FP clinics Kenya (the FP Plus project, Clinical Trial.Gov: NCT04666792). The study details and parent protocol are described elsewhere (7). Briefly, the FP Plus Project was a pragmatic stepped wedge cluster-randomized project to integrate systematic screening and offer oral PrEP to women accessing twelve primary care FP clinics in Kenya.”*

*In addition, to improve clarity, we revised the sentences in the study strengths in lines 313-316 as follows:*

*“Our samples of 1800 women were drawn from the general population of women who visited the 12 FP clinics participating in the large programmatic cluster randomized trial during the 24-months. There are also limitations to our study. First, this was a cross-sectional study prone to the inherent flaws of this exploratory design.”*

*Thank you for this important comment. The study did not collect data on reasons why a few women were dissatisfied with their clinic experience. Notably, overwhelmingly women (>98%) expressed satisfaction for both clinic experience and trust in healthcare providers. Very often health providers in this setting are not given the due recognition they deserve as we tend to only focus on the negative reported attributes. This often ignores the context in which these providers work, including an overburdened health system and high client volumes. Thus, although we agree and recognize the important value in identifying modifiable reasons for nonsatisfaction, we respectfully believe it is more appropriate to highlight the overwhelming enthusiasm. Importantly, our complementary qualitative interviews in parent study will help us provide useful insights into the underlying reasons for stated clinic experience positive or otherwise.*

*We added the following sentence in the discussion in lines 276-279 “Very often health providers in this setting are not given the due recognition they deserve as we tend to only focus on the negative reported attributes. This often ignores the context in which these providers work, including an overburdened health system and high client volumes.”*

What theoretical framework informed measurement of patient satisfaction? We thank the reviewer for this excellent comment. Although the survey documented itself and we have added it as a key limitation, all the items/questions on the survey were collated from psychometrically developed tools and validated for this setting. Importantly, we have used this tool and items in this setting and population in several of our multiple clinical and implementation studies (including the Partners Scale up in HIV care clinics, PrIYA program in MCH/FP) and are confident in its content validity*. We have added the following sentences under limitations in lines 318-323.*

*“**Secondly, although the survey documented itself, all its items were derived from psychometrically developed instruments that have been validated for use in this setting. Importantly, we have previously employed this tool and its components in several clinical and implementation studies within the same population, such as the Partners Scale-Up in HIV care clinics and the PrIYA program in MCH/FP, which strengthens our confidence in its content validity”*

**Reviewer #2:**
Novelty & Context
• Congratulations to the research team for highlighting the novelty of real-world family planning (FP) clinic data from Kenya. This contribution is valuable for understanding HIV prevention opportunities in routine service delivery settings.

Study Period
• Line 35 states the study period as June 2021 – April 2024, whereas line 95 lists July 2021 – July 2024. Please align these dates and specify exact months to ensure clarity and reproducibility.

*Response. We thank the reviewer for this valuable suggestion. We apologize for the inconsistency. The study period was July 2021 – July 2024. We have revised the manuscript in lines 32-34 as follows.*

*“Between July 26, 2021, and 07/July 07, 2024, we conducted a cross-sectional study nested within a large pragmatic trial of PrEP delivery integrated in twelve real-world FP clinics in Kisumu Kenya (Clinical Trials.Gov: NCT04666792).”*

Data Presentation & Quantification
• Line 49: Self-perceived risk and reasons for PrEP decline are summarized qualitatively but not fully quantified, limiting interpretive power. Consider including proportions or counts to strengthen the impact of this section.

*Response: We thank the reviewer for this valuable suggestion. We have revised by presenting the percentages in lines 44-46 as currently seen below:*

*“One-third (30.6%, 551/1801) of this population were assessed to have elevated HIV risk and eligible for PrEP per guidelines, but over half of them (72.2%, 1300/1801)) expressed low self-perceived HIV risk and declined PrEP.”*

*In addition, based on your additional review below, we decided to revise the entire manuscript by presenting frequencies and proportions, as appropriate.*

• Lines 51, 54, 55, 201, 207: Several metrics lack precision. Examples include “>69%” for oral PrEP knowledge, “>98” for trust in providers without a stated scale, and “>80%” for FP clinics as the preferred access point. Please use exact percentages (or clearly defined approximate ranges) and specify measurement scales (e.g., Likert) to improve statistical validity and reproducibility.

*Response: We thank the reviewer for this valuable suggestion. We have revised the entire manuscript by presenting precise metrics as counts and corresponding percentages.*

Terminology Consistency
• Minor: Lines 41 and 191 use “findings” and “results” interchangeably. For coherence, choose one term and apply consistently throughout.

*Response: We thank the reviewer for this valuable review. We have updated by adhering to the PLOS Global Health submission guidelines. The revised version uses the term results as seen in line 199.*

Denominators & Missing Data
• Lines 217, 232, 235, 236 report denominators that vary substantially (e.g., condom use n=505 in line 217 vs. n=5512 — larger than the total sample size of 1801). Please clarify how missing data were handled (e.g., exclusion, imputation) and use consistent denominators or justify differences.

*Response: We thank the reviewer for this valuable review and suggestion. We are sorry for the confusion caused on previous line 217. The n was 551 and not 5512. We have revised as seen currently in line 247.*

*We used a complete case analysis because of its simplicity, and for most of the variables we had complete data.*

Subgroup Analyses
• The results do not explore urban–rural or clinic-type differences despite the diversity of sites. Consider adding a subgroup or stratified analysis, even as a supplementary table, to provide contextual nuance and inform targeted interventions.

*Response: We thank the reviewer for this invaluable comment. We have now added clinic location variable categorized as urban, peri-urban, rural) in Tables 1 and 3. Additionally, we have also added a table with demographics and general characteristics of the study population stratified by clinic location as a supplementary file.*

Discussion Enhancements
• Comparative references to Uganda and South Africa are relevant but dated (pre-2024). Consider citing recent 2024–2025 studies on long-acting PrEP (e.g., cabotegravir trials) to highlight the contemporary relevance of the Kenya findings.

*Response: We thank the reviewer for this valuable suggestion. We added the following language to the discussion in lines 301-310 as follows*

*“**This study was conducted in the context with only oral PrEP as the available standard of care PrEP option. Although long-acting injectable cabotegravir and dapivirine vaginal ring are already approved in Kenya, but they are yet available as standard of care in public health clinics. Lenacapavir is still under regulatory review in Kenya but ap it readily available as standard of care in the short-term. A recent demonstration project in Uganda and Kenya that offered a person-centered intervention with choice of delivery point and PrEP products, including long-acting injectable cabotegravir improved HIV prevention coverage by more than 50% compared to standard of care. Taken together, this demonstrates the tremendous potential to expand coverage and the reach for new users if choice and access to these emerging multiple options with improved availability and equitable access”*

• Ethical implications, particularly stigma in partner consultation, are underexplored. Adding a paragraph on stigma mitigation strategies and ethical safeguards would strengthen this section and align with human *rights-based approaches. We thank the reviewer for this important comment, and we totally agree on the stigma in partner consultation. For this cross-sectional survey, however, we did not explore in detail stigma and partner influence, and we believe it would be only speculation to comment on them. We have acknowledged this as a limitation for this report. Importantly, in the large parent study we conducted complementary qualitative interviews with women which will help provide in-depth insights on these contextually important themes on stigma and partner influence.*

*We added a sentence in the limitations on lines 323-327 as follows:*

*“Thirdly, for this cross-sectional survey, however, we did not examine stigma and partner influence in detail, and therefore it would be speculative to draw conclusions regarding these factors. Importantly, within the larger parent study, we conducted complementary qualitative interviews with women, which will provide deeper insights into these contextually important themes of stigma and partner influence.”*

Conclusion & Forward-Looking Recommendations
• While the conclusion effectively summarizes findings and emphasizes the importance of FP clinics in HIV prevention, it largely repeats the discussion. Strengthen this section by adding forward-looking, actionable recommendations—such as scalable provider training models, differentiated service delivery approaches, or digital tools to support adherence—while emphasizing ethical and equitable scale-up for underserved women.

*Response: We thank you for this valuable suggestion. We revised the conclusion*

*The revised conclusion section is presented in lines 332-339 as follows:*

*“**In summary, in this large cross-sectional study among women accessing high-volume real-world FP clinics in Kenya, we have shown that FP clinics represent a preferred and trusted platform for integrating and expanding HIV prevention services for women in Africa settings. The findings show that FP clinics are uniquely positioned to serve as key access points for women who may benefit from comprehensive healthcare services, including PrEP for HIV prevention. Future research should focus on addressing gaps in HIV risk perception, knowledge of sexual partner status, and PrEP awareness with targeted interventions and defining efficient delivery models integrating emerging long-acting PrEP options in real-world settings.”*
