# Supplementary material for: Population characteristics, PrEP eligibility, and trust in family planning providers among women accessing public family planning clinics in Kenya
Source: PLOS Glob Public Health. 2026 Feb 26;6(2):e0005480. doi: 10.1371/journal.pgph.0005480 (PMC12944804; doi:10.1371/journal.pgph.0005480)
Supplement: Attachment — Submitted filename: Response_to_Editorial_Team_and_Reviewers_auresp_2.docx [file pgph.0005480.s004.docx]

Dr. Andrew Kazibwe

Academic Editor

PLOS Global Public Health

RE: Submission ID: [PGPH-D-25-02465] - [EMID:ea28ca1fa7b6ae4b]

Dear Dr Andrew, Kazibwe,

On behalf of my co-authors, we are grateful for your valuable suggestions and recommendations and for the opportunity to revise and improve our study. Our manuscript has benefitted tremendously from these comments.

Below, we have attached a point-by-point response to each review suggestion, comment, and recommendation in *Italics*.

Thank you for allowing us to resubmit our manuscript for your consideration.

We look forward to hearing from you.

Sincerely,

David Mukasa, BVM, MVPM, PhD, MS, FRSPH

Assistant Teaching Professor, Department of Global Health

School of Public Health and School of Medicine

University of Washington

3980 15th Ave NE, Seattle, WA 98105

Phone: +1 (206) 685-2329

Email: [mukasa@uw.edu](mailto:mukasa@uw.edu)

**Response to Editorial Team and Reviewers**

If the reviewer comments include a recommendation to cite specific previously published works, please review and evaluate these publications to determine whether they are relevant and should be cited. There is no requirement to cite these works unless the editor has indicated otherwise.

***Response****:* *We did not cite any articles because of recommendations from reviewers.*

Please review your reference list to ensure that it is complete and correct. If you have cited papers that have been retracted, please include the rationale for doing so in the manuscript text, or remove these references and replace them with relevant current references. Any changes to the reference list should be mentioned in the rebuttal letter that accompanies your revised manuscript. If you need to cite a retracted article, indicate the article’s retracted status in the References list and also include a citation and full reference for the retraction notice.

***Response:*** We reviewed all references to ensure they are complete, accurate, current, and appropriately support the statements made in the manuscript. None of the cited references have been retracted, and no retraction notices are required.

Additional Editor Comments (if provided):

This is a well written manuscript with a current topic

***Response:*** *We thank the reviewer for this positive assessment and appreciate the thoughtful and constructive feedback that has greatly helped us to improve our manuscript and the additional feedback provided below.*

Line 118 Population of Kisumu County?

***Response:*** *Thank you for this comment. We have clarified the population context of Kisumu County in the revised manuscript to provide geographic and demographic background relevant to the study setting.*

*We have revised the manuscript in lines 118-120 as follows:*

*“The study was conducted in Kisumu County, Kenya, a region with a population of approximately 1.15 million people and an HIV prevalence of 15.6% among the adult population and up to 22% among women”*

Line 190 … “informed” consent…Also specify how <18 gave consent and why it is allowed in this context

***Response:*** *Thank you for this comment. Participants under 18 years of age who were pregnant were emancipated and provided their own informed consent, as the study was minimal risk and involved reproductive health research. Parental permission and assent were therefore not required.*

Line 202 Women enrolled 1801, sample size determination was 1639. Make a note of why? E. g., attrition rate consideration?

***Response:*** *Thank you for noting this. The calculated sample size represented the minimum required to achieve adequate statistical power. To account for an anticipated non-response or exclusion rate of approximately 10%, we planned to enroll a larger number of participants, resulting in a total enrollment of 1,801 women. This has now been clarified in the Methods section.*

*We added the following sentence in lines 173-174:*

*“A total of 1801 women were enrolled to account for potential 10% non-response rate.”.*

Line 278 Did you probe for the fear of reprisal that could influence response?

***Response:*** *Thank you for raising this point. Fear of reprisal was not directly probed through specific survey items. However, the study incorporated procedures intended to minimize this risk, including conducting interviews in private settings, using trained interviewers, and assuring participants of confidentiality. We believe there is no reason to doubt the results of the outcome.*

Line 284 …opportunity to “know” how…

***Response:*** *Thank you so much for the meticulous review. We have revised the sentence to improve clarity and precision.*

*We have updated by adding the word “understand” as seen in line 286.*

*“Identifying characteristics of women accessing public sector FP clinics offers an opportunity to understand how to target and expand comprehensive healthcare services”*

Line 304 Remove “but”

***Response:*** *Thank you for noting this. The word “but” has been removed to improve sentence flow.*

Line 305 What is “ap”?

***Response:*** *Thank you for identifying this. The unintended abbreviation has been removed, and the sentence has been revised to improve clarity, but also the drug has been approved as of 2026 and not under review as we had previously written. To reflect these revisions and updates, we have revised the sentence in lines as seen below:*

*“Lenacapavir received regulatory approval in Kenya in early 2026; however, widespread availability as standard of care will depend on national rollout, guideline inclusion, and implementation timelines.”*

Line 317, 319 ‘Secondly’ twice

***Response:*** *Thank you for the valuable feedback. The repetition has been corrected in the revised manuscript, and the limitations are now presented in the correct chronological sequence.*

Line 319-320 Explain how this is a limitation

***Response:*** *Thank you for this comment. We have clarified how this represents a limitation by explaining that, although individual survey items were derived from psychometrically developed instruments validated for use in this setting, the survey did not administer the full original instruments. As a result, assessment of the psychometric properties of the combined survey was limited, and comparability with studies using the complete instruments may be reduced. This clarification has been incorporated into the revised version.*

*We revised as follows:*

*“Thirdly, although survey items were derived from psychometrically developed instruments validated for use in this setting, the survey did not administer the full original instruments, which may limit assessment of psychometric properties and comparability with prior studies”*

Table 1 Were there any specific reasons for dissatisfaction in overall clinical experience?
***Response:*** *Thank you for this question. While overall satisfaction was assessed, specific reasons for dissatisfaction were not systematically captured in the survey. As a result, these could not be examined in detail.*

Reviewer #2: Congratulations for addressing all comments in detail. Please ensure the line spacing from 312 to 331 are in sync with the other sections. Congrats once again to all the authors.

***Response:*** *Thank you for noting this formatting issue. Line spacing has been corrected to ensure consistency with the rest of the manuscript.*

*We sincerely thank the editorial team and the reviewers for their thoughtful and constructive feedback, which has strengthened the clarity and overall quality of the manuscript.*
